# Right upper lobe segmentectomy and subsegmentectomy guided by classification pattern of peripheral segmental veins

**DOI:** 10.3389/fonc.2023.1179570

**Published:** 2023-09-06

**Authors:** Zhikai Li, Shuangqing Chen, Dahu Ren, Yuhong Kong, Shun Xu, Guochen Duan, Xiaopeng Zhang

**Affiliations:** ^1^Graduate School, Hebei Medical University, Shijiazhuang, China; ^2^Department of Thoracic Surgery, Hebei General Hospital, Shijiazhuang, China; ^3^Graduate School, Hebei North University, Zhangjiakou, China; ^4^Department of Thoracic Surgery, The First Hospital of China Medical University, Shenyang, Liaoning, China; ^5^Department of Thoracic Surgery, Children’s Hospital of Hebei Province, Shijiazhuang, China

**Keywords:** right upper lobe (RUL), vein variations, classification, identification approach, segmentectomy, lobectomy, non-small cell lung carcinoma (NSCLC), video-assisted thoracoscopic surgery (VATS)

## Abstract

**Background:**

Studies have analyzed the simplified branching pattern of peripheral segmental veins and developed a standardized approach for intersegmental vein identification in the right upper lobe (RUL). However, the identification approach of intersubsegmental veins has not been reported. This study aimed to supplement the identification approach of intersubsegmental veins and the classification pattern of peripheral segmental veins by using three-dimensional computed tomography bronchography and angiography (3D-CTBA).

**Materials and methods:**

A total of 600 patients with ground glass opacity (GGO) who had undergone 3D-CTBA preoperatively at Hebei General Hospital between September 2020 and September 2022 were used for the retrospective study. We reviewed the anatomical variations of RUL veins in these patients using 3D-CTBA images.

**Results:**

According to the anatomical position, the peripheral segmental veins structures of RUL were classified into five categories: “Iab type of anterior with central vein” (256/600, 42.7%), “Ib type of anterior with central vein” (166/600, 27.7%), “Central vein type” (38/600, 6.3%), “Anterior vein type” (81/600, 13.5%), “Right top pulmonary vein type” (57/600, 9.5%). The approach for intersegmental vein and intersubsegmental veins identification was divided into five types: anterior approach, posterobronchial approach, central vein approach, V^2^t approach, and intermediate bronchus posterior surface approach.

**Conclusions:**

The classification pattern of peripheral segmental veins should find wide application. Further, approaches identifying intersegmental veins and intersubsegmental veins may help thoracic surgeons perform safe and accurate RUL segmentectomy.

## Introduction

Anatomical segmentectomy, which preserves more lung function and minimizes lung volume loss, is increasingly used in treating early-stage non-small cell lung carcinoma (NSCLC). Several studies have indicated the same oncologic efficacy between a video-assisted thoracoscopic surgery (VATS) lobectomy and segmentectomy (1–5). However, a segmentectomy is more challenging than a standard lobectomy because of the anatomical variation of the peripheral vessels and bronchi. In addition, it is challenging to recognize pulmonary veins variants from conventional two-dimensional (2D) CT images. Recently, three-dimensional computed tomography bronchography and angiography (3D-CTBA), which extracts high-quality planar image data from CT scans and creates three-dimensional (3D) virtual models of the lungs, including segments, subsegments, lesions, bronchi, and vessels, is a useful tool for thoracic surgeons to identify intersegmental veins and intersubsegmental veins more intuitively compared with conventional 2D-CT images. Thus, general thoracic surgeons can more easily perform safe and accurate anatomical segmentectomy with the aid of 3D-CTBA.

The pulmonary segmental veins is an essential anatomical marker for anatomical segmentectomy. Similar to how intersegmental veins travel between adjacent lung segments and serve as the natural demarcation of lung segments, intersubsegmental veins also move between adjacent subsegments and serve as the natural demarcation of lung subsegments. Successful anatomical segmentectomy requires intraoperative identification of intersegmental veins and intersubsegmental veins. Therefore, it places a higher demand on the thoracic surgeon, including a comprehensive understanding of the pulmonary bronchovascular pattern, especially the typical and uncommon anatomical variants of branching patterns of pulmonary veins.

In recent years, only a few systematic reports describing variations in the right upper lobe (RUL) vein patterns have been published (6–8). However, these reports do not adequately categorize the peripheral segmental veins. The purpose of the present study was to supplement the classification of peripheral segmental veins in the RUL by using data derived from 3D-CTBA and compare the results of our study with those of similar study that was previously conducted (7). Moreover, we adopt innovative methods for identifying the intersegmental veins and intersubsegmental veins according to the model subtype to plan safe RUL segmentectomy and avoid pulmonary vein injuries.

## Materials and methods

### Patient preparation

The chest-enhanced CT examinations preoperatively were implemented on 600 patients with ground glass opacity (GGO) at Hebei General Hospital from September 2020 to September 2022. All procedures involving human participants in this study were in accordance with the Declaration of Helsinki (revised in 2013). This retrospective study was approved by the Research Ethics Committee at Hebei General Hospital (No. 2022119), and informed consent was waived from all patients.

### Reconstruction of 3D-CTBA

We performed preoperative chest-enhanced CT by using Siemens 64-slice dual-source CT (Somatom Definition) with the contrast agent as ioversol 350. A total of 70 mL contrast medium (ioversol 350) was administered intravenously at a rate of 2-3 mL/s. Contrast-enhanced CT was performed using fixed time method. The arterial phase scans were taken 30s after contrast injection and the venous phase scans 90s after contrast injection. By setting a scan start time, the CT values of the pulmonary veins and arteries revealed density variations in the images. The patients were required to hold their breath throughout the CT scan for appropriate bronchial inflate, and precautions were taken to avoid any potential side effects from the contrast agent following the scan. The volume data from both arterial and venous phases were imported into a reconstruction software (Infer Operate Thorax Planning), which computed and processed the data before presenting it in 3D-CTBA images.

### Definition of pulmonary vein

**Figure 1:**
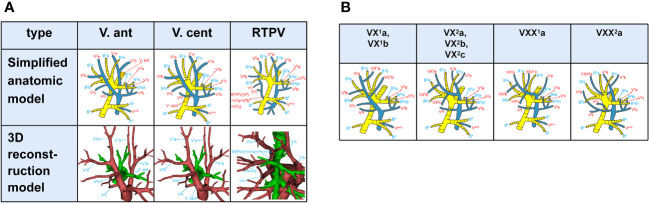
**(A)** Simplified anatomic model and 3D reconstruction model of V. ant, V. cent, and RTPV. **(B)** Simplified anatomic model of VX^1^a, VX^1^b, VX^2^a, VX^2^b, VX^2^c, VXX^1^a, and VXX^2^a.

According to the nomenclature principle of Boyden, Nagashima, and Miyamoto (6, 7, 9, 10), branching of the pulmonary vein was classified into the following three types (**Figure 1A**): the anterior vein (V. ant), the central vein (V. cent) and the right top pulmonary vein (RTPV). V. ant (**Figure 1A**), arising from V^1^b and going down the anterior side of the RUL bronchus, ultimately flows into the superior pulmonary vein (SPV) from the mediastinal side (7). V. cent (**Figure 1A**), originating from V^2^a and descending between B^2^ and B^3^, finally flows into the SPV from the interlobar side (7). RTPV (**Figure 1A**), usually originating from the S^2^ and descending the posterior surface of the intermediate bronchus, is an anomalous vein (10–12).

V^1^a, V^2^b, and V^3^a moving between subsegments were defined as intersubsegmental veins, while V^1^b, V^2^a, and V^2^c running between segments as intersegmental veins (**Figure 1A**). Basically, V^1^a flows into V^1^b from the mediastinal side and V^2^ into V. cent or V^2^t. Thus, when V^1^ flows into V. cent, and V^2^ flows into V. ant (7), the intersegmental are called VX (VX^1^b, VX^2^c, **Figure 1B**) and the intersubsegmental veins are called VX (VX^1^a, VX^2^b, **Figure 1B**); when V^1^a flows out of the center of S^1^ and into V^1^b between B^1^ and B^3^, we named it as VXX^1^a (**Figure 1B**). Likewise, when V^2^a drains into V. ant, it was defined as follows based on anatomical position (7): A, VX^2^a moves through the center of the RUL between B^1^ and B^3^ (**Figure 1B**); B, VXX^2^a moves on the mediastinal surface of S^1^ (**Figure 1B**).

## Results

### Peripheral segmental veins structures of RUL

**Figure 2:**
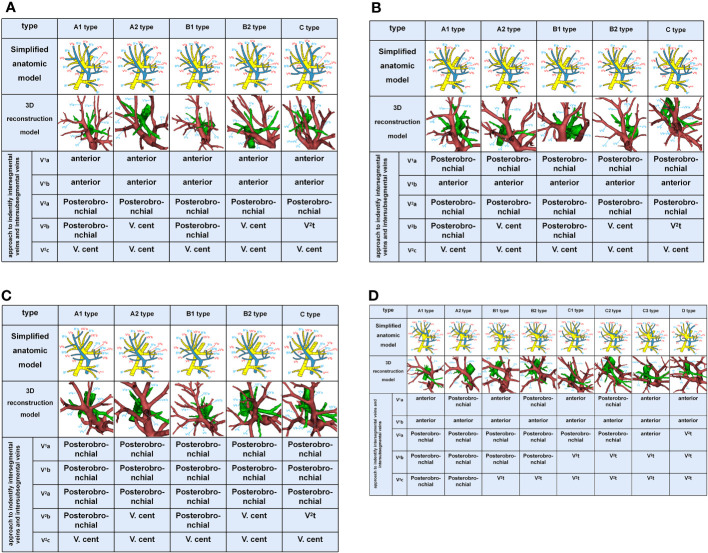
**(A)** Simplified anatomic model and 3D reconstruction model of Iab type and the recommended approach to indentify intersegmental vein (V^1^b, V^2^a, and V^2^c) and intersubsegmental veins (V^1^a and V^2^b). **(B)** Simplified anatomic model and 3D reconstruction model of Ib type and the recommended approach to identify intersegmental vein (V^1^b, V^2^a, and V^2^c) and intersubsegmental veins (V^1^a and V^2^b). **(C)** Simplified anatomic model and 3D reconstruction model of Central vein type and the recommended approach to identify intersegmental vein (V^1^b, V^2^a, and V^2^c) and intersubsegmental veins (V^1^a and V^2^b). **(D)** Simplified anatomic model and 3D reconstruction model of Anterior vein type and the recommended approach to identify intersegmental vein (V^1^b, V^2^a, and V^2^c) and intersubsegmental veins (V^1^a and V^2^b).

**Figure 3:**
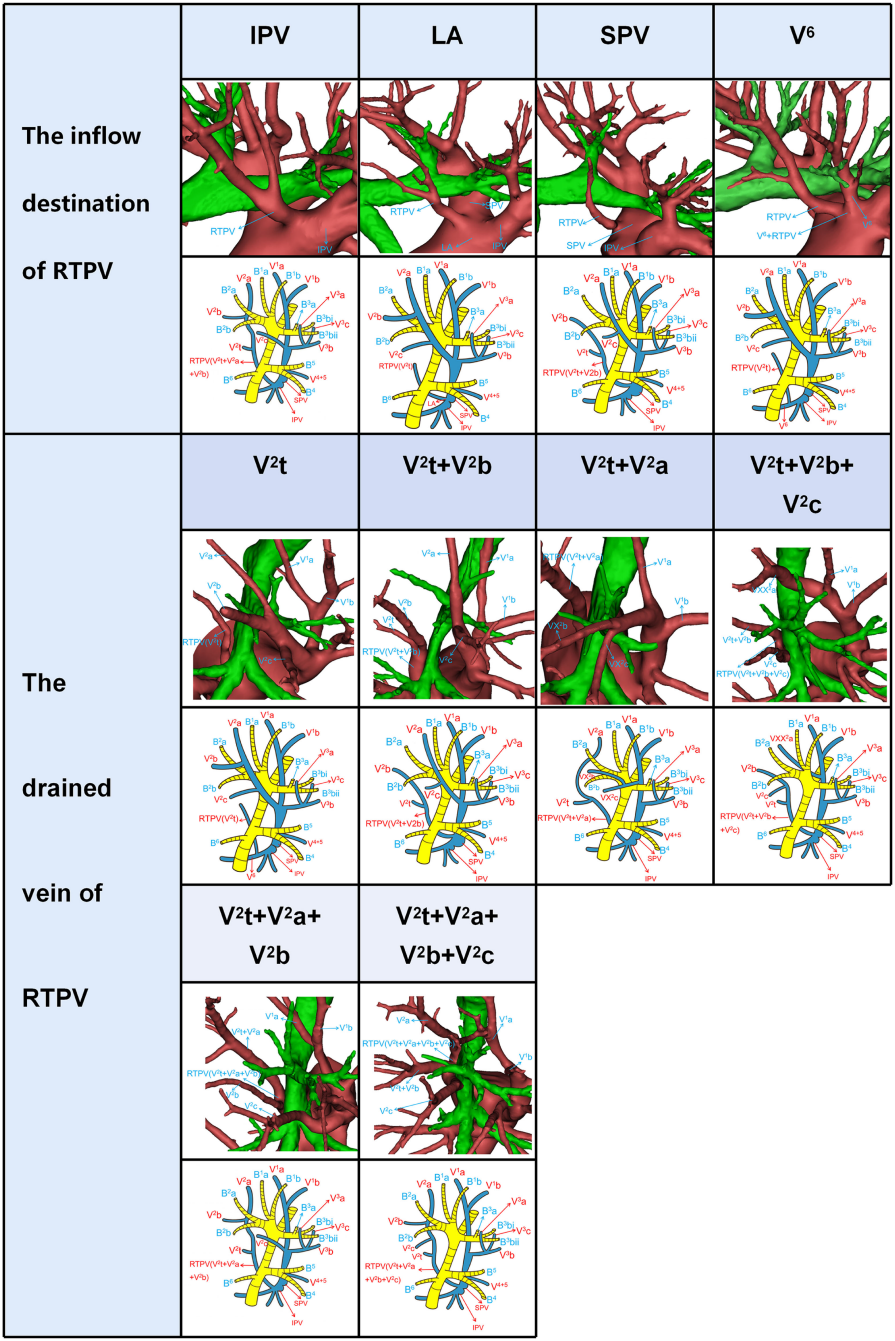
Simplified anatomic model and 3D reconstruction model of the inflow destination of RTPV and the drained vein of RTPV.

**Figure 4:**
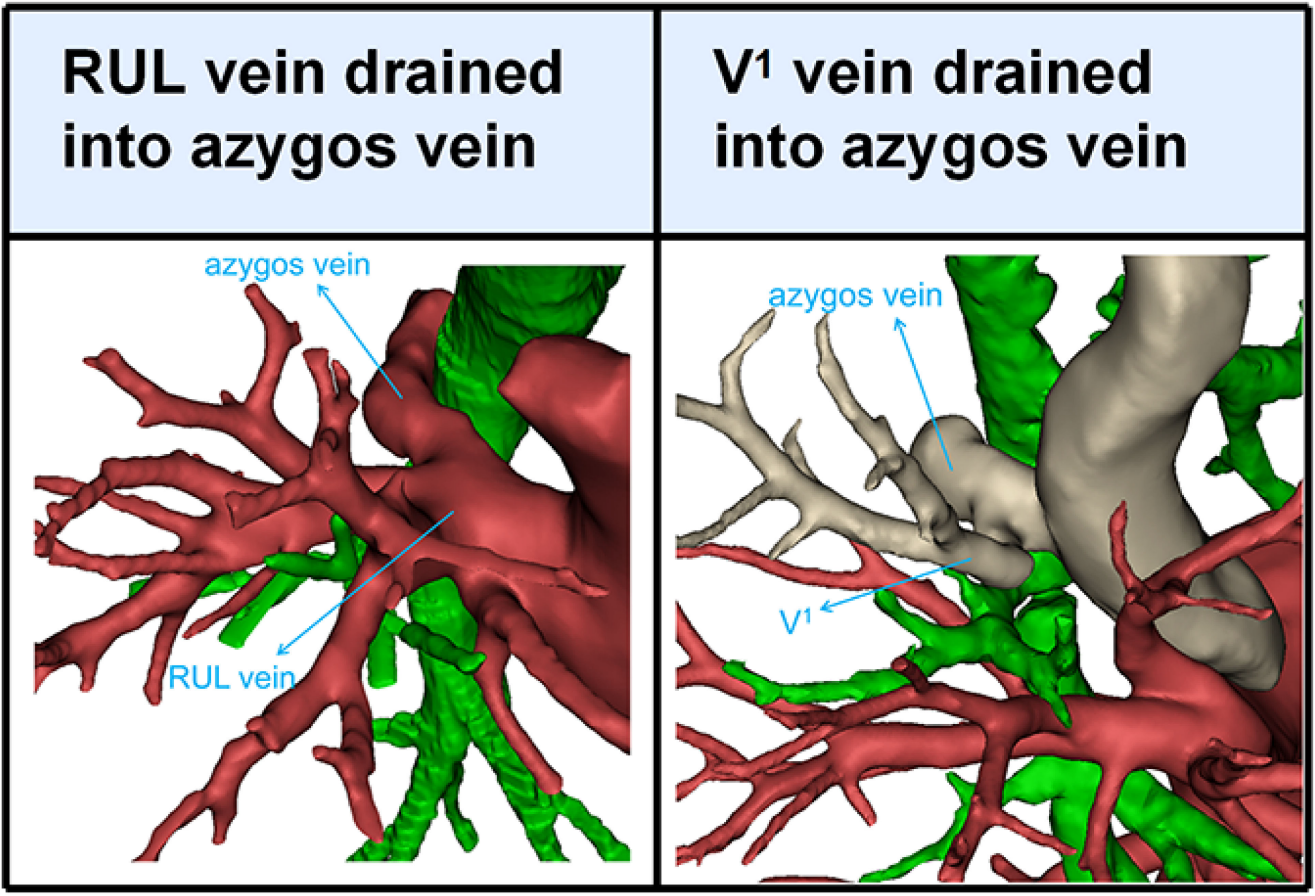
3D reconstruction model of variation of drainage in the azygos vein type.

Branching of the pulmonary vein of RUL was divided into five types (**Table 1**; **Figures 2A–D**, **3**): “Iab type of anterior with central vein” (256/600, 42.7%), “Ib type of anterior with central vein” (166/600, 27.7%), “Central vein type” (38/600, 6.3%), “Anterior vein type” (81/600, 13.5%), “Right top pulmonary vein type” (57/600, 9.5%). Two cases with rare variations were excluded from our classification system, including one case that V^1^ drained into azygos vein and another case that RUL vein drained into azygos vein (**Figure 4**).

**Table 1:** The peripheral segmental veins patterns of five types.

	Our study (n=600)		Nagashima (n=338)	
	NO.	%	NO.	%
Iab type	256	42.7	184	54
A1 type	163	64	132	72
A2 type	20	8	NR	–
B1 type	9	3	13	7
B2 type	16	6	NR	–
C type	48	19	28	15
Other	NR	–	11	6
Ib type	166	27.7	89	26
A1 type	127	77	67	75
A2 type	9	5	NR	–
B1 type	7	4	7	8
B2 type	8	5	NR	–
C type	15	9	8	9
Other	NR	–	7	8
Central vein type	38	6.3	23	7
A1 type	20	53	14	61
A2 type	5	13	NR	–
B1 type	3	8	3	13
B2 type	3	8	NR	–
C type	7	18	2	9
Other	NR	–	4	17
Anterior vein type	81	13.5	42	13
A1 type	7	9	10	24
A2 type	11	13	NR	–
B1 type	5	6	6	14
B2 type	5	6	NR	–
C1 type	15	19	13	31
C2 type	10	12	NR	–
C3 type	13	16	12	29
D type	15	19	NR	–
Other	NR	–	1	2
RTPV	57	9.5	NR	–
N/A	2	0.3	NR	–

N/A, not available; NR, the type was not referred.

V. ant originates from V^1^a and V^1^b in Iab type (**Figure 2A**). Similarly, V. ant arises from only V^1^b in Ib type (**Figure 2B**). However, V. ant is absent in Central vein type (**Figure 2C**). Each of these three types (Iab, Ib, and Central) can be further classified into five anatomical categories (A1, A2, B1, B2, and C subtype) based on the branching pattern of the central vein (**Table 1**). In A type, V^2^b and V^2^c each drain independently into V^2^a. And A type was further subclassified into two types according to the location of V^2^b: A1 subtype, V^2^b runs above B^2^b (**Figures 2A-C**); A2 subtype, V^2^b runs below B^2^b (**Figures 2A-C**). In B type, a common trunk sharing V^2^b with V^2^c drains into V^2^a. Likewise, B type was defined as follows based on the location of V^2^b: B1 subtype, V^2^b runs above B^2^b (**Figures 2A–C**); B2 subtype, V^2^b runs below B^2^b (**Figures 2A-C**). In C type (**Figures 2A-C**), a common trunk sharing V^2^t with V^2^b drains into V^2^a at a central location to V^2^c. Since V^3^ is not anatomically involved in lung segmental structures except for V^3^a, V^3^ veins were not further classified in the present study.

In the Anterior vein type, V^1^ (V^1^a and V^1^b)+V^2^ (V^2^a, V^2^b, and V^2^c) drains into V. ant and V^2^t, whereas V. cent is lacking (**Figure 2D**). It was classified into four types (A, B, C, D subtype) according to V^1^+V^2^ (**Table 1**, **Figure 2D**). The common trunk of VX^2^a+VX^2^b+VX^2^c drains into V. ant in A type. The common trunk of VX^2^a+VX^2^b drains into V. ant and the V^2^c drains into V^2^t in B type. The common trunk of V^2^b+V^2^c drains into V^2^t in C type. The common trunk of V^2^a+V^2^b+V^2^c drains into V^2^t in D type. The A type and B type were respectively subclassified into two subtypes according to the anatomical position of V^1^a. The V^1^a drains into V. ant in A1 and B1 subtype, while the VXX^1^a drains into V. ant in A2 and B2 subtype (**Figure 2D**). The C type was further subclassified into three subtypes by reference to the position of V^1^a and V^2^a (**Figure 2D**): C1 subtype, a common trunk of V^1^a+VX^2^a drains into V. ant; C2 subtype, a common trunk of VXX^1^a+VX^2^a drains into V. ant; C3 subtype, a common trunk of V^1^a+VXX^2^a drains into V. ant. In the D type, a common trunk of V^2^a+V^2^b+V^2^c drains into V^2^t and V^1^a drains into V. ant (**Figure 2D**).

RTPV was classified into four subtypes by reference to the inflow destination and six types according to the vein drained (**Table 2**, **Figure 3**). Moreover, RTPV finally drains into the inferior pulmonary vein (IPV), the left atrium (LA), SPV, or V^6^.

**Table 2:** Summary of RTPV.

	Our study (n=600)		Shiina (n=189)	
	NO.	%	NO.	%
The inflow destination of RTPV				
IPV	22	3.7	7	3.7
LA	21	3.5	2	1.1
SPV	6	1.0	2	1.1
V^6^	8	1.3	3	1.6
The drained vein of RTPV				
V^2^t	31	5.2	NR	–
V^2^t+V^2^b	11	1.8	NR	–
V^2^t+V^2^a	3	0.5	NR	–
V^2^t+V^2^b+V^2^c	5	0.8	NR	–
V^2^t+V^2^a+V^2^b	4	0.7	NR	–
V^2^t+V^2^a+V^2^b+V^2^c	3	0.5	NR	–

### Approaches identifying intersegmental veins and intersubsegmental veins

**Figure 5:**
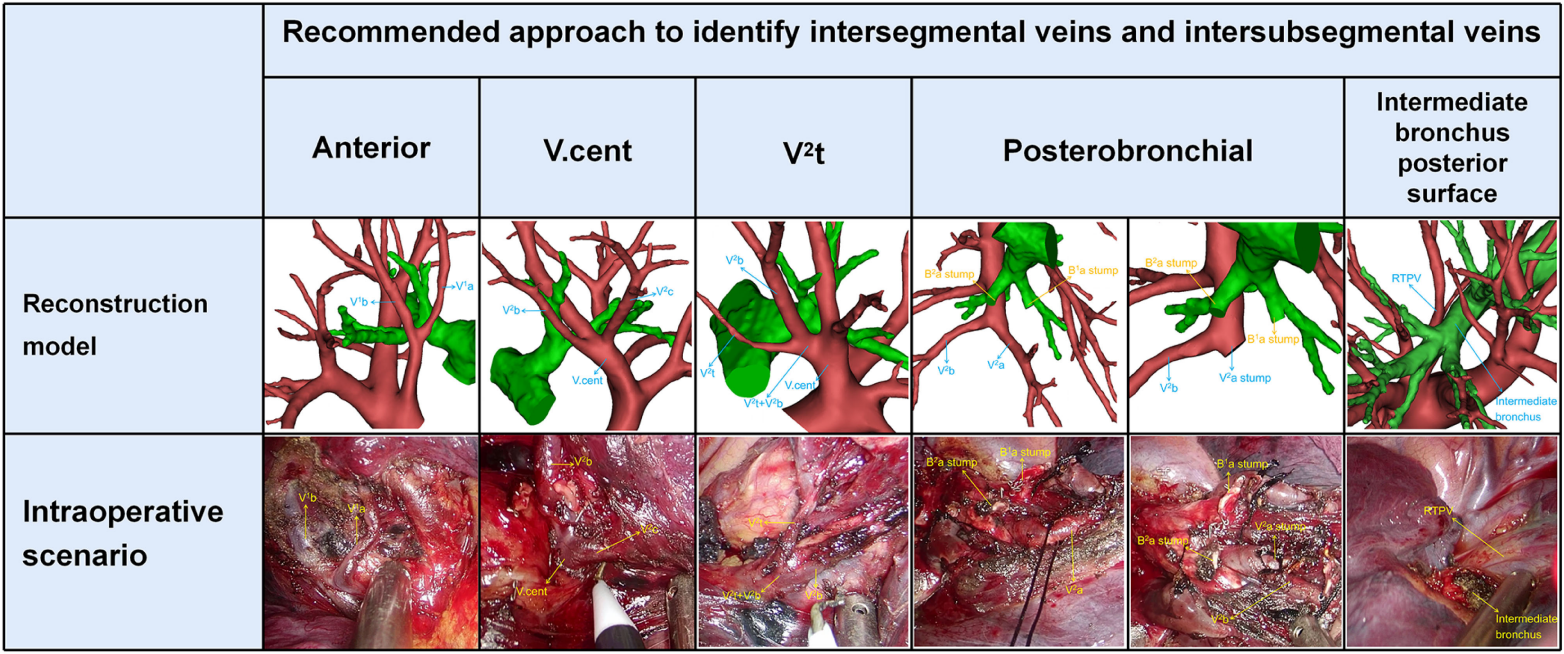
Five basic approaches to identify intersegmental veins and intersubsegmental veins. Anterior approach: identification of intersegmental vein (V^1^b) and intersubsegmental veins (V^1^a) by dissecting along mediastinal surface of S^1^ in the S^1^ segmentectomy. V.cent approach: identification of intersegmental vein (V^2^c) and intersubsegmental veins (V^2^b) by dissecting along V.cent in the S^2^ segmentectomy. V^2^t approach: identification of intersubsegmental veins (V^2^b) by dissecting along V^2^t in the S^2^ segmentectomy. Posterobronchial approach: identification of intersegmental vein (V^2^a) and intersubsegmental veins (V^2^b) which are located posterior to the bronchus, usually after dissection of the targeted bronchus (B^1^a, B^2^a) in the S^1^a+S^2^a segmentectomy. Intermediate bronchus posterior surface approach: identification of RTPV by dissecting along the intermediate bronchus posterior surface in S^2^ segmentectomy.

Anterior approach was adopted to distinguish veins which move on the mediastinal surface of S^1^ (**Figure 5**). Similarly, V. cent approach was used to identify veins which run through the lung parenchyma surface of S^2^ (**Figure 5**). In addition, the V^2^t approach was used to dissect veins draining into V^2^t (**Figure 5**). Posterobronchial approach was applied to dissect veins running deep within the lung parenchyma (**Figure 5**). Intermediate bronchus posterior surface approach was utilized to dissect RTPV (**Figure 5**).

## Discussion

With the application of high-resolution computed tomography (HRCT), the detection rate of GGO increases. Some research results have shown that the prognosis of segmentectomy is no worse than that of lobectomy in patients with early lung cancer (1–5). The JCOG0802 study is a randomized, controlled, non-inferiority trial to confirm whether segmentectomy is not inferior to lobectomy regarding prognosis (1). The JCOG0802 study illustrated segmentectomy to be non-inferior and superior to lobectomy with regards to overall survival and concluded segmentectomy should be the standard surgical procedure, rather than lobectomy, for patients with small-sized (≤2 cm, consolidation-to-tumour ratio >0.5) peripheral non-small cell lung carcinoma (NSCLC) (1). CALGB 140503 study indicated that in patients with peripheral NSCLC with a tumor size of 2 cm or less and pathologically confirmed node-negative disease in the hilar and mediastinal lymph nodes, sublobar resection was not inferior to lobectomy with respect to disease free survival. In addition, sublobar resection provides valuable surgical opportunities for patients who could not undergo standard lobectomy because of the limited cardiopulmonary reserve. In order to perform sublobar resection successfully, the thoracic surgeon must have a thorough understanding of the spatial anatomy of the intersegmental veins and intersubsegmental veins.

This classification system focuses on the intersegmental veins and intersubsegmental veins for two reasons. First, the intersegmental veins and intersubsegmental veins are structures that physically define the boundaries of the segments and subsegments, and thus they are applied to the preoperative schema to determine whether the surgical margin between the tumor and segmental border or subsegmental border is sufficient. Second, dissection of the intersegmental plane or intersubsegmental plane at the hilum is usually done along these intersegmental veins and intersubsegmental veins.

In this study, the Iab type was seen in 42.7% of cases (**Table 1**), which was lower than the frequency reported by Nagashima (7) (54%). The Ib type and Central vein type were respectively seen in 27.7% and 6.3% of cases, which was the same as the frequency reported by Nagashima, 26% and 7% (7). We found that the Iab type, Ib type and Central vein type had respectively five subtypes. In addition, the structure of A1, B1, and C subtype are the same as that of Nagashima (**Figures 2A–C**). However, the structure of A2, B2 subtype has not been reported in the literature (**Figures 2A–C**). The main reason is the spatial relationships of V^2^b and B^2^b.

Nagashima found the Anterior vein type accounted for 13%, which is similar to our results (**Table 1**). And the Anterior vein type was divided into eight subtypes in our study (**Figure 2D**). Moreover, we found that the structure of A1, B1, C1, and C3 subtype are the same as that of Nagashima (7). However, the structure of A2, B2, C2 subtype has not been reported in Nagashima’s s study (**Figure 2D**). The main reason is that V^1^a runs deeping within the lung parenchyma of S^1^. In addition, the D type also has not been found in Nagashima’s s study owing to the draining vein of V^2^t (**Figure 2D**).

Shiina’s s study has reported the classification of the inflow destination of RTPV (**Table 2**, **Figure 3**), which is the same as our results (10). And IPV is the most common type for the inflow destination of RTPV. However, we found the draining vein of RTPV has six subtypes (**Table 2**, **Figure 3**), which have not been reported in the literature.

Nakazawa’s study recommended three basic approaches to identify intersegmental veins (13). In the present study, the approaches to identify V^1^b are the same as in Nakazawa’s study (**Table 3**). However, the approaches to identify V^2^a and V^2^c are different to Nakazawa’s study (**Table 3**). In addition, the approaches to identify intersubsegmental veins, which are crucial to subsegmentectomy have not been reported in the previous study. We supplement the approaches to identify V^1^a and V^2^b (**Table 3**).

**Table 3:** Recommended approach to identify intersegmental or intersubsegmental veins.

	V^1^a	V^1^b	V^2^a	V^2^b	V^2^c
anterior	Iab type Anterior vein type(A1,B1,C1,C3,D)	Iab type Ib type Anterior vein type	Anterior vein type(C3)	–	–
Posterobro-nchial	Ib type Central vein type Anterior vein type(A2,B2,C2)	Central vein type	Iab type Ib type Central vein type Anterior vein type(A1,A2, B1,B2,C1,C2)	Iab type(A1,B1) Ib type(A1,B1) Central vein type(A1,B1) Anterior vein type(A1,A2,B1,B2)	Anterior vein type (A1,A2)
V. cent	–	–	–	Iab type(A2,B2) Ib type(A2,B2) Central vein type(A2,B2)	Iab type Ib type Central vein type
V2t	–	–	Anterior vein type (D)	Iab type(C) Ib type(C) Central vein type(C) Anterior vein type(C1,C2,C3,D)	Anterior vein type(B1,B2,C1,C2,C3,D)
Intermedia-te bronchus posterior surface	–	–	RTPV	RTPV	RTPV

Different approaches were used in patients with anatomic variants in their intersegmental veins and intersubsegmental veins (**Table 3**, **Figure 5**). V^1^a and V^1^b always move on the mediastinal surface of S^1^ or deep within the lung parenchyma of S^1^. Therefore, V^1^a can usually be identified by anterior approach except for Ib type, central vein type, and Anterior type (A2, B2, C2), for which a posterobronchial approach is necessary (**Table 3**, **Figures 2A–D**). Similarly, V^1^b can be distinguished by anterior approach except for the Central vein type, for which a posterobronchial approach is necessary (**Table 3**, **Figures 2A–D**).

V^2^a usually runs deep within the lung parenchyma among S^1^a and S^2^a, whereas it can also move on the mediastinal surface of S^1^ or drains into V^2^t or RTPV (**Table 3**, **Figures 2A–D**, **3**). Therefore, V^2^a can be dissected by Posterobronchial approach except for the Anterior vein type (C3, D subtype) and RTPV type. Moreover, V^2^a can also be identified through the anterior approach for the Anterior vein type (C3 subtype), or V^2^t approach for the Anterior vein type (D subtype), or the intermediate bronchus posterior surface approach for RTPV type (**Table 3**, **Figures 2A–D**, **3**).

V^2^c usually runs the lung parenchyma surface of S^2^, whereas it can also runs deep within the lung parenchyma among S^1^ and S^3^ or drains into V^2^t or RTPV. Therefore, V^2^c can be identified by the V.cent approach except for the Anterior vein type and RTPV type (**Table 3**, **Figures 2A–D**, **3**). In addition, V^2^c can also be distinguished through Posterobronchial approach for the Anterior vein type (A1 and A2 subtype), or V^2^t approach for the Anterior vein type (B1, B2, C1, C2, C3, and D subtype), or intermediate bronchus posterior surface approach for RTPV type (**Table 3**, **Figures 2A–D**, **3**).

Compared to V^1^a, V^1^b, V^2^a and V^2^c, peripheral segmental vein types related to approaches identifying V^2^b shows more diversity. V^2^b can run deep within the lung parenchyma among S^2^ or the lung parenchyma surface of S^2^, whereas it can also drain into V^2^t or RTPV. For Iab type (A1 and B1 subtype), Ib type (A1 and B1 subtype), Central vein type (A1 and B1 subtype), and Anterior vein type (A1, A2, B1, and B2 subtype), V^2^b runs deep within the lung parenchyma among S^2^ and therefore identified by Posterobronchial approach (**Table 3**, **Figures 2A–D**). For Iab type (A2 and B2 subtype), Ib type (A2 and B2 subtype), and Central vein type (A2 and B2 subtype), V^2^b moves on the lung parenchyma surface of S^2^ and consequently distinguished through the V.cent approach (**Table 3**, **Figures 2A–C**). For Iab type (C subtype), Ib type (C subtype), Central vein type (C subtype), and Anterior vein type (C1, C2, C3, and D subtype), V^2^b can be dissected by V^2^t approach owing to it drains into V^2^t (**Table 3**, **Figures 2A–D**). Similarly, For the RTPV type, V^2^b can be dissected by the RTPV approach owing to it draining into RTPV (**Table 3**, **Figure 3**).

Finally, it is vital to master RTPV for safe lobectomy. The previous study has shown an increased incidence of incomplete fissures in patients with RTPV (11). Thus, when the incomplete fissure is discovered intraoperatively, it is crucial to keep an eye on the existence of the RTPV. It is easy to cause bleeding and air leakage of the lung by splitting the lung fissure abruptly. At this time, we can utilize the modified inflation-deflation methods to determine the location of the lung fissure. In the RUL lobectomy, if RTPV is present, we should apply appropriate traction to the RUL in order to avoid damage to RTPV. In right lower lobe (RLL) lobectomy, if the RTPV is damaged while it drains into the IPV or V^6^, infarction and necrosis of the RUL might occur. Moreover, it is important to the prognosis of primary lung cancer in the right middle lobe (RML) and RLL by dissecting lymph nodes at the tracheal bifurcation. Therefore, in RML or RLL lobectomy, the inflow destination of RTPV should be considered during lymphadenectomy. In conclusion, RTPV is a common type of venous anomaly, and we should understand it accurately.

This study had several limitations. Firstly, the segmental artery and the bronchi were excluded from models, which promote understand peripheral segmental veins. However, variations in segmental veins are closely associated with those of the segmental bronchi and arteries. Secondly, we only use 3D-CTBA data, which may differ from the intraoperative anatomical findings.

## Conclusions

This study illustrates that converting the RUL anatomy into simplified anatomic models helps thoracic surgeons understand the classification of peripheral segmental veins. Further, approaches to identifying intersegmental veins and intersubsegmental veins provide certain references that surgeons can use to plan and perform RUL segmentectomy.

## Data availability statement

The original contributions presented in the study are included in the article/supplementary material. Further inquiries can be directed to the corresponding authors.

## Ethics statement

This retrospective study was approved by the Research Ethics Committee at Hebei General Hospital (no. 2022119). The need for patient consent was waived because of the retrospective nature of the study.

## Author contributions

ZL: project design and initiation, data analysis, manuscript writing. YK: project design and initiation, data analysis, manuscript writing. DR: project design and initiation, data analysis, manuscript writing. SC: data collection. XZ: supervisor. All authors contributed to the article and approved the submitted version.
